# Investigating the Role of microRNA in Host–Influenza A and Other Respiratory RNA Virus Interactions

**DOI:** 10.3390/pathogens15090889

**Published:** 2026-08-25

**Authors:** Carla Prezioso, Flavio Frezza, Stefano Aquaro, Lucia Nencioni, Annaluisa Mariconda, Diana Amantea, Alessia Catalano, Pasquale Longo, Maria Stefania Sinicropi, Paola Checconi

**Affiliations:** 1 Department for the Promotion of Human Sciences and Quality of Life, San Raffaele University, Via di Val Cannuta 247, 00166 Rome, Italy; carla.prezioso@uniroma5.it (C.P.); flavio.frezza@uniroma5.it (F.F.); 2 Laboratory of Microbiology, IRCCS San Raffaele Roma, Via di Val Cannuta 247, 00166 Rome, Italy; 3 Department of Life, Health and Environmental Sciences, University of L’Aquila, Piazzale Salvatore Tommasi, 1, Blocco 11, Coppito, 67010 L’Aquila, Italy; stefano.aquaro@univaq.it; 4 Department of Public Health and Infectious Diseases, Laboratory Affiliated to Istituto Pasteur Italia-Fondazione Cenci Bolognetti, Sapienza University, 00185 Rome, Italy; lucia.nencioni@uniroma1.it; 5 Department of Basic and Applied Sciences, University of Basilicata, Via dell’Ateneo Lucano, 10, 85100 Potenza, Italy; annaluisa.mariconda@unibas.it; 6 Department of Pharmacy, Health and Nutritional Sciences, University of Calabria, Via Pietro Bucci, 87036 Arcavacata di Rende, Italys.sinicropi@unical.it (M.S.S.); 7 Department of Pharmacy-Drug Sciences, University of Bari “Aldo Moro”, Via Orabona, 4, 70126 Bari, Italy; 8 Department of Chemistry and Biology “A. Zambelli”, University of Salerno, Via Giovanni Paolo II, 132, 84084 Salerno, Italy; plongo@unisa.it

**Keywords:** microRNAs, influenza A virus, respiratory RNA viruses, miRNA biogenesis, antiviral immunity, viral immune evasion, viral immunopathology, biomarkers, miRNA-based therapeutics

## Abstract

Respiratory RNA viruses extensively reprogram host regulatory networks, thereby influencing viral replication, immune evasion, and disease severity. This review examines microRNAs (miRNAs) as regulatory interfaces in host–virus interactions, focusing on influenza A virus as a paradigmatic model while integrating evidence from other respiratory RNA viruses as SARS-CoV-2 and respiratory syncytial virus. After outlining canonical miRNA biogenesis and its manipulation during infection, we discuss how respiratory RNA viruses converge on shared miRNA-regulated pathways, including interferon and NF-κB signaling, apoptosis, autophagy, cellular metabolism, and redox homeostasis. Within these networks, host miRNAs can directly target viral RNAs and modulate antiviral defenses and inflammation, whereas viruses can reshape miRNA expression to facilitate replication, influencing immunopathology. The possibility that RNA viruses encode authentic miRNAs is also critically evaluated; current evidence indicates that manipulating host miRNA biogenesis machinery and remodeling miRNA networks are more prevalent than producing canonical viral miRNAs. Finally, the potential of circulating miRNAs as diagnostic and prognostic biomarkers is considered, as well as the capability of miRNA mimics and antagomiRs to function as host-directed therapeutic strategies. Their clinical translation, however, will require standardized validation, cell- and time-resolved studies, efficient delivery systems, and a careful assessment of specificity, safety, and context-dependent effects.

## 1. Introduction

Virus infections represent a major challenge to host defense systems, as these pathogens rely extensively on cellular machinery to complete their replication cycle and to remodel the intracellular environment in favor of viral propagation. Over the past decades, it has become increasingly evident that, beyond classical protein–protein interactions, viruses modulate complex host regulatory networks, including those mediated by microRNAs (miRNAs), which play a central role in the pathology of several respiratory viral infections [1,2]. MiRNAs are small non-coding RNAs that regulate gene expression at the post-transcriptional level, thus contributing to the regulation of numerous biological processes, including development, cell proliferation, apoptosis, immunity, and inflammatory responses [3,4,5]. MiRNAs are now recognized as a critical layer of gene regulation during viral infections. Under physiological conditions, they contribute to the maintenance of cellular homeostasis; however, during infection, their expression profiles can be profoundly altered as a consequence of host immune sensing or direct viral manipulation. Such remodeling of the miRNA landscape influences multiple aspects of viral pathogenesis, including pathogen recognition, interferon (IFN) production, NF-κB activation, pro-inflammatory cytokine secretion, autophagy, and programmed cell death. Consequently, miRNAs should not be viewed merely as accessory components of the antiviral response, but rather as central regulatory nodes at the interface between virus and host [6,7].

Unlike many DNA viruses, for which the production of virus-encoded miRNAs is well documented [8], RNA viruses appear to primarily modulate host miRNAs, rather than encoding their own miRNA through host miRNA biogenesis machinery, to minimize the risk of RNA genome processing and degradation [9,10]. As a result, the outcome of RNA virus infection often depends on the ability of the virus to reprogram pre-existing host regulatory circuits. In this context, host miRNAs may exert opposing effects: some are rapidly induced as part of the innate antiviral response and contribute to restricting viral replication, whereas others are exploited by viruses to suppress immune defenses, promote cell survival, or establish conditions favorable for viral replication [11].

Among RNA viruses, respiratory viruses such as influenza A virus (IAV) represent some of the most extensively studied models for investigating host miRNA–virus interactions. IAV is responsible for seasonal epidemics and recurrent pandemics associated with substantial morbidity and mortality worldwide [12]. IAV has evolved sophisticated mechanisms to modulate host-cell pathways [13]. During infection, airway cells undergo extensive transcriptional and post-transcriptional reprogramming, involving numerous miRNAs also [2,14]. Several studies have demonstrated that specific host miRNAs can directly target viral transcripts or cellular factors required for efficient IAV replication, thereby limiting viral spread [15,16]. Conversely, IAV can alter the expression of host miRNAs to modulate key signaling pathways, ultimately promoting immune evasion and ensure viral replication [14,17]. Beyond their role in regulating viral replication, miRNAs also contribute to the immunopathology associated with influenza infection. Dysregulation of specific miRNAs has been linked to virulence in IAV pandemic strain infections [18], as well as to hyperinflammatory responses, cytokine storm development, acute lung injury, and acute respiratory distress syndrome (ARDS) [19], suggesting that miRNAs also influence disease severity and clinical outcome. However, although the involvement of miRNAs in DNA virus infections has been extensively characterized, and much has also been described about RNA viruses, many aspects of miRNA regulation during RNA virus infections remain incompletely understood. In particular, the mechanisms through which RNA viruses remodel host miRNA networks and converge on common host signaling pathways require further investigation. Moreover, the existence of authentic virus-encoded miRNAs in RNA viruses remains controversial, owing to the presence of non-canonical viral small RNAs and the technical challenges associated with their identification and functional validation. In light of these observations, this review examines the role of miRNAs in host–respiratory RNA virus interactions, focusing on IAV as a paradigmatic, primary model of host miRNA network remodeling during infection, and integrating selected evidence from severe acute respiratory syndrome coronavirus 2 (SARS-CoV-2) and respiratory syncytial virus (RSV) to identify shared and virus-specific miRNA-regulated pathways. We discuss how miRNAs function as shared regulatory interfaces between virus and host, influencing viral replication, innate immune responses, inflammation, and infection-associated immunopathology. Finally, we consider the diagnostic and therapeutic potential of miRNAs in the context of respiratory RNA viral infections. Literature databases (PubMed/MEDLINE, Scopus, and Google Scholar) were used as sources to search the literature. General keywords such as “miRNA”, “Influenza A virus”, and “Respiratory viruses” were used. All abstracts and full-text articles were examined for their relevance to this review.

## 2. Host miRNA Biogenesis and Its Modulation During Viral Infection

### 2.1. Canonical miRNA Biogenesis and Its Functional Role

The canonical miRNA biogenesis pathway provides the molecular framework through which viruses can subsequently influence host gene expression. Because many RNA viruses alter miRNA abundance, maturation, or activity to facilitate infection, understanding the biochemical and molecular bases of miRNA biogenesis and action is essential for interpreting the mechanisms underlying virus–host interactions [20,21]. In the canonical pathway, miRNAs are generated through a tightly regulated multistep process involving both nuclear and cytoplasmic compartments. MiRNA genes are transcribed primarily by RNA polymerase II, producing long primary transcripts (pri-miRNAs) that contain one or more characteristic stem–loop structures. Similar to protein-coding transcripts, pri-miRNAs are typically capped and polyadenylated before undergoing further processing [22]. Within the nucleus, pri-miRNAs are recognized and cleaved by the microprocessor complex, composed of the RNase III enzyme Drosha and its essential cofactor DGCR8 [23]. This initial processing step generates precursor miRNAs (pre-miRNAs), approximately 70 nucleotides in length, which retain their characteristic hairpin structure. The pre-miRNAs are subsequently exported to the cytoplasm by Exportin-5 in a Ran-GTP-dependent manner, thereby linking nuclear processing to cytoplasmic maturation [24]. In the cytoplasm, pre-miRNAs are further processed by Dicer, a second RNase III enzyme that removes the terminal loop of the hairpin and generates a double-stranded RNA of approximately 22 nucleotides [25]. This duplex is loaded onto an Argonaute (AGO) protein (AGO1—4), predominantly AGO2 [26], and consists of a guide strand, which is retained, and a passenger strand, which is degraded. In fact, once incorporated with an AGO protein, the duplex is separated and the passenger strand is expelled, leading to the formation of the active RNA-induced silencing complex (RISC), the effector machinery responsible for miRNA-mediated gene silencing [27]. Once within the RISC, mature miRNAs guide sequence-specific recognition of target mRNAs, primarily through partial complementarity with sequences located within the 3′-untranslated regions (3′-UTRs), even if, recently, alternative target recognition mechanisms have been also described [28]. Depending on the degree of complementarity and the cellular context, miRNA binding can result in gene silencing through translational repression, mRNA destabilization, or deadenylation and decay, thereby fine-tuning gene expression at the post-transcriptional level [29]. A notable exception to these negative regulation mechanisms has been reported for miR-122 and HCV, as this miRNA has been shown to mediate viral RNA stabilization and translational activation [30,31]. This kind of RNA interference is evolutionary conserved throughout eukaryotes [32] and commonly functions to defend hosts against harmful RNA, such as those from viruses [33]. In fact, host miRNAs, although different from other small non-coding RNAs in their biogenesis, which occurs as described above, are nonetheless considered part of cell defense machinery through the broad phenomenon known as RNA silencing [34]. During viral infection, host cells rapidly remodel their miRNA repertoire in response to viral sensing and the activation of innate immune pathways, and as a result, numerous miRNAs function to limit viral replication and propagation through complementary mechanisms [35,36]. One mechanism involves the direct targeting of viral RNA molecules. By binding complementary sequences within viral genomes or transcripts, host miRNAs can reduce viral gene expression, impair genome replication, and ultimately limit the production of infectious particles. Song et al. (2010) [15] demonstrated that miR-323, miR-491, and miR-654 bind to the influenza A virus H1N1 *PB1* gene and downregulate *PB1* expression through mRNA degradation, thus inhibiting IAV replication. Peng et al. (2018) [16] identified four other miRNA-targeting viral gene segments of the human seasonal strain H3N2. Other miRNAs have been reported to directly target viral RNA as well, thus suppressing RNA viral replication [37,38]. Through computational analyses and molecular docking simulations, hsa-miR-2278 and hsa-miR-6732-3p have been predicted to bind to RSV mRNA and to represent potential transcriptional inhibitors [37]. The antiviral efficacy of this process depends on several factors, including the accessibility of target sites and the number of miRNA-binding sequences present within viral RNAs. Consequently, direct miRNA-mediated restriction represents a sequence-specific layer of host defense that may substantially influence viral fitness [39,40]. In addition to direct antiviral activity, many host miRNAs exert their effects indirectly through the modulation of host factors required for the viral replication cycle, such as cellular receptors that can alter viral adsorption and tropism [40]. A transcriptomic study on lung epithelial cells infected with SARS-CoV-2 revealed altered miRNA expression profiling upon infection; among the upregulated miRNAs, miR-1246 has been shown to decrease the mRNA of angiotensin-converting enzyme 2 (ACE2), the major viral cell-entry receptor [41]. Another study demonstrated that transforming growth factor (TGF)-β1 inhibited ACE2 expression by a miRNA-mediated mechanism, which may decrease SARS-CoV-2 cell entry too [42]; moreover, TGF- β1 may exert other miRNA-mediated effects on immune response. In fact, miRNAs act indirectly also through the modulation of innate immune signaling pathways. Upon infection, viral pathogen-associated molecular patterns (PAMPs) are detected by pattern-recognition receptors (PRRs), including retinoic acid inducible gene-I (RIG-I) and Toll-like receptors (TLRs). The activation of these sensors triggers signaling cascades involving IFN regulatory factors (IRF3 and IRF7) and NF-κB, ultimately resulting in the production of type I interferon (IFN-I) and pro-inflammatory cytokines [43]. In particular, increasing evidence indicates that miRNAs play a central role in regulating the RIG-I receptor/interferon axis, promoting the expression of interferon-stimulated genes (*ISGs*), which constitute one of the major antiviral defense mechanisms, thereby reinforcing the antiviral state of infected and neighboring cells [44,45,46]. Among these, miR-26a and miR-30a for instance have emerged as potent positive regulators of type I IFN responses. By targeting Ubiquitin-Specific Peptidase 15 (*USP15)* and *USP14*, respectively, they promote a RIG-I-mediated interferon axis, ultimately restricting viral replication [47,48]. Moreover, the same authors showed that type I IFN responses suppress miR-26a expression in turn to avoid a disordered activation that could result in the so-called “type I interferonopathy”, highlighting regulatory feedback loops that fine-tune the extent of antiviral responses [47]. Recent advances in high-throughput sequencing, RNA-RNA, and RNA–protein interaction mapping have considerably expanded our understanding of miRNA biogenesis and functions, revealing additional regulatory checkpoints that influence miRNA maturation, target recognition, and turnover [21,49,50]. Because each step of this pathway depends on tightly regulated host factors, the miRNAs’ biogenesis machinery and their target mRNAs provide multiple potential targets for viral interference [51]. Indeed, accumulating evidence indicates that respiratory RNA viruses actively manipulate components of the miRNA processing pathway to reshape host gene expression programs, attenuate antiviral responses, and create conditions favorable for viral replication [52]. The mechanisms underlying these viral perturbations are discussed in the following Section 2.2.

### 2.2. Viral Manipulation of the miRNA Machinery

**Figure 1:**
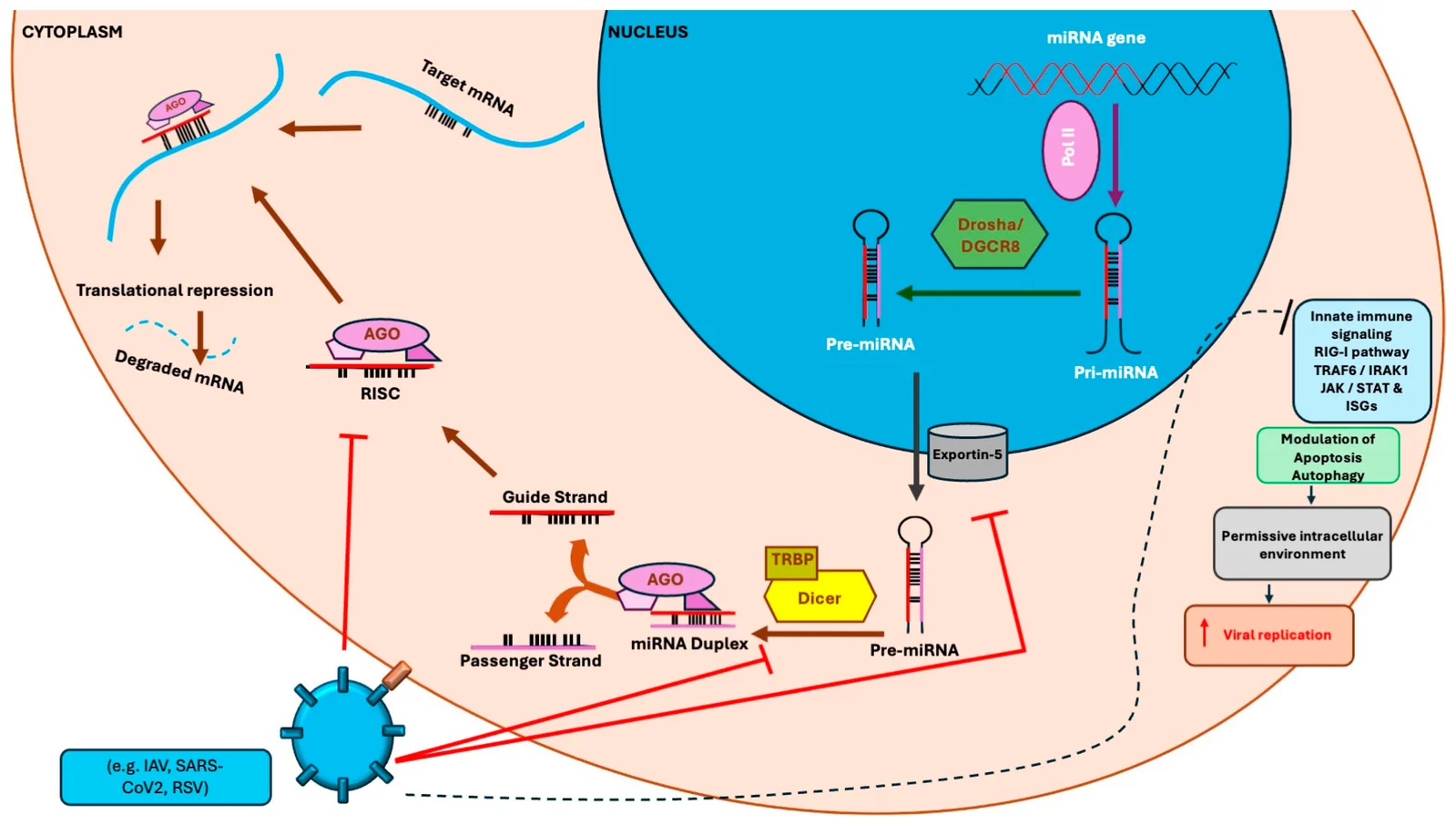
Viral strategies to manipulate host miRNA machinery. Solid arrows indicate the direction of the represented biological processes; red lines indicate viral inhibition or interference with specific steps of the miRNA pathway and the dashed line indicates the downstream consequences of viral modulation.

The canonical miRNA biogenesis pathway is highly dependent on host regulatory factors and therefore represents an attractive target for viral manipulation (Figure 1). While the modulation of individual miRNAs constitutes an important component of host–virus interactions, increasing evidence suggests that many RNA viruses act at a higher regulatory level by targeting the cellular machinery responsible for miRNA biogenesis and function. Such a strategy enables viruses to influence multiple miRNA networks simultaneously, thereby amplifying their impact on host gene expression, antiviral defense, and inflammatory responses [52,53]. In this context, pro-viral miRNAs are generally defined as host-derived miRNAs whose expression or activity ultimately benefits the virus by attenuating antiviral responses, modulating cellular homeostasis, or promoting the survival of infected cells [40]. Importantly, most pro-viral miRNAs are not inherently beneficial to viruses. Rather, they are physiological regulators of immune responses and cellular homeostasis whose activity can be co-opted by viral pathogens under specific infection contexts. This concept highlights the dual nature of miRNA-mediated regulation and emphasizes that the biological outcome of miRNA activity often depends on timing, cellular environment, and infection stage [54]. Thus, RNA viruses have evolved mechanisms to directly interfere with components of miRNA processing machinery. Although the extent of such interactions remains incompletely understood, accumulating evidence indicates that viral components can modulate the activity of key factors involved in miRNA biogenesis, including Drosha, Dicer, Argonaute proteins, and their associated cofactors [21]. One of the first-reported proteins was the HIV Tat protein, which has been shown to inhibit Dicer activity, thus reducing the maturation of miRNAs [55]. Later, Flaviviruses, including Dengue virus, have been described to suppress miRNA production through non-coding subgenomic viral RNAs that associate with Dicer and AGO2 in cultured cells and mosquitoes, the vector of the virus [56]. Among RNA respiratory viruses, one of the best-characterized examples of direct interference with miRNA machinery has been described for the IAV. Li et al. (2016) [57] showed that human Dicer mediated the generation of viral small-interfering RNA from IAV double-stranded RNA precursors in infected cells; these small interfering RNAs were strongly inhibited by IAV non-structural protein NS1, thus reporting both the induction and suppression of antiviral RNA interference during IAV infection. Then, it was shown that viral NS1 interacts with the TAR RNA-binding protein (TRBP), a double-stranded RNA-binding cofactor that associates with Dicer and facilitates the processing of precursor miRNAs into mature miRNAs. Through this interaction, NS1 impairs TRBP-dependent RNA interference activity and reduces the efficiency of miRNA maturation, highlighting how viral proteins can directly target host post-transcriptional regulatory pathways to favor infection [58]. Regarding SARS-CoV-2, it has been shown that NSP2 interacts with AGO2 via GIGYF2 and enhances the translational repression mediated by natural miRNA-binding sites in the 3′ untranslated region of cellular mRNAs, revealing an additional layer of the complex mechanism by which a virus and likely other coronaviruses manipulate the host gene expression program by co-opting host miRNA-mediated silencing machinery [59]. Moreover, it has been shown that the SARS-CoV-2 N protein induces the autophagic degradation of Exportin-5 and Dicer, and this was correlated with the severity of viral pneumonia [60]. Interestingly, not all RNA viruses appear to suppress miRNA biogenesis. Emerging evidence suggests that some viruses preserve or selectively exploit components of the host miRNA machinery. Transcriptomic analyses performed during SARS-CoV-2 infection revealed sustained expression of several genes involved in miRNA biogenesis, including *DROSHA*, *DICER1*, *DGCR8*, *AGO2*, and *XPO5*, suggesting that viral adaptation may involve selective remodeling rather than the complete inhibition of the pathway [61]. These observations support the notion that the viral manipulation of miRNA pathways encompasses a spectrum of strategies ranging from pathway suppression to selective exploitation of host regulatory networks. Another principal mechanism through which proviral miRNAs support infection involves the suppression of innate immune signaling pathways [39]. Effective antiviral responses depend on the rapid activation of PRR, including the aforementioned RIG-I-mediated signaling, type I IFN production, and expression of *ISGs*. Several host miRNAs target components of these pathways, generating regulatory feedback loops that normally function to prevent excessive immune activation. However, viruses can exploit these same regulatory circuits to dampen antiviral responses and establish a more permissive intracellular environment [11]. One of the first studies on this topic demonstrated that miR-146a, upregulated during vesicular stomatitis virus (VSV) infection, is a negative regulator of the RIG-I-dependent antiviral pathway by targeting *TRAF6* and *IRAK1* and *IRAK2*, thus promoting viral replication in macrophages [62]. Later on, it was shown that the expression of miR-485, induced in response to infections with various other RNA viruses, including the influenza virus, targeted RIG-I mRNA for degradation, leading to the suppression of the antiviral response and enhancing viral replication [63]. Subsequent studies expanded the list of host miRNAs induced by viral infections, which targets RIG-I itself, including miR-92a, miR-218 [64,65], or components of the downstream signaling pathway, such as miR-340, miR-200b [66,67], and miR-203a, which interfere with JAK/STAT and *ISGs* expression, facilitating RNA virus replication [68]. Beyond IFN signaling, proviral effects may also arise through the modulation of programmed cell death and apoptosis, a fundamental host defense mechanism that limits viral dissemination through the elimination of infected cells [69,70]. By altering host miRNA expression, viral infections can shift the balance between pro-apoptotic and anti-apoptotic pathways, creating a more permissive environment for viral replication [71]. Virus-induced changes in miRNA expression have also been implicated in the regulation of autophagy and other cellular stress-response pathways, highlighting the broad impact of miRNA-mediated regulation on virus–host interactions [69]. Collectively, these findings indicate that antiviral miRNAs function not only by directly targeting viral RNAs but also by orchestrating host defense pathways. Through the integration of post-transcriptional regulation and innate immune signaling, host miRNAs provide an additional layer of protection against viral infection and contribute significantly to the outcome of host–pathogen interactions [39,70]. Conversely, RNA viruses try to evade miRNA-mediated regulation actively exploiting miRNA networks to manipulate host cellular responses. By co-opting regulatory pathways that control immunity, apoptosis, and cellular homeostasis, viruses can reshape the intracellular environment in ways that promote viral replication [40,69,70]. Importantly, the manipulation of miRNA processing machinery enables viruses to exert regulatory effects that extend far beyond the modulation of single miRNAs; rather, they can target the maturation and activity of hundreds of miRNAs inducing widespread changes in cellular gene expression [11,21]. Such global rewiring of miRNA networks may affect IFN signaling, inflammatory responses, apoptosis, autophagy, and cellular metabolism, processes that we explore in detail in the following Section 3, focusing on respiratory RNA viruses.

## 3. Common miRNA-Regulated Pathways Across Respiratory RNA Viruses

Despite substantial differences in genome organization, replication strategies, tissue tropism, and pathogenicity, respiratory RNA viruses frequently converge on conserved host pathways regulated by miRNAs and involved in antiviral immunity, inflammation, apoptosis, autophagy, and metabolic homeostasis. This convergence highlights the central role of host miRNAs as regulatory hubs at the interface between viral infection and cellular responses (Table 1). Consequently, understanding these shared pathways may provide broader insights into respiratory viral pathogenesis and reveal common targets for therapeutic intervention.

### 3.1. IFN-I Signaling as a Central Hub

The IFN-I signaling pathway represents one of the most important innate immune mechanisms activated following RNA virus infection. Upon recognition of viral RNA by PRRs, including RIG-I and TLRs, signaling cascades culminate in the activation of IRFs (IRF3 and IRF7) and subsequent production of IFN-I. Secreted IFNs then activate the JAK/STAT pathway and induce the expression of hundreds of *ISGs*, establishing a potent antiviral state [89]. Host miRNAs can either enhance or suppress IFN-I pathway, thus promoting (or not) antiviral immune responses, through targeting different key signaling intermediates [45]. As a result, the IFN-I pathway has emerged as one of the principal points of convergence across diverse respiratory RNA viruses [90,91,92]. Moreover, the relationship between IFN signaling and miRNA biology is bidirectional. In fact, if on the one hand, numerous miRNAs target components of the IFN signaling cascade [45,93], on the other hand, IFN stimulation itself induces the expression of miRNAs, generating regulatory feedback loops that fine-tune the magnitude and duration of antiviral responses [94,95,96]. During IAV infection, several miRNAs have been described to modulate antiviral responses. For instance, miR-9-1 and miR-206 have been shown to target two poly(ADP-ribose) polymerases, tankyrases 1 and 2, inducing IFN responses and thus decreasing IAV replication [72,73]; the miR-302-IRFs axis facilitated the transcription of key hub genes and lncRNAs, most of which significantly reduced IAV replication [74]; and recently, avian miR-92 was shown to enhance IFN-I signaling by directly targeting *TNFRSF1B*, thereby limiting the *TNFRSF1B*-mediated autophagolysosomal degradation of TRAF3 and inhibiting IAV replication. This antiviral effect was observed against both H9N2 and H1N1 viruses in avian DF-1 cells, whereas in human A549 cells, miR-92 inhibited H1N1 but not H9N2 replication [77]. Among miRNAs that have shown a pro-viral effect, miR-146a and miR-200c have been reported to enhance IAV replication [75,76], while miR-141 downregulated antiviral genes such as *MxA* to ensure viral replication [17]. Negative regulators of IFN signaling, as members of the suppressor of the cytokine signaling (SOCS) family, also represent recurrent targets of miRNA-mediated control: IAV downregulated miR-221, which targeted *SOCS1*; as consequences, the *SOCS1* level increased and IFN-I responses were suppressed [77]. In SARS-CoV-2 infection, dysregulated miRNA expression profiles have been also associated with impaired IFN responses and pathogenesis [42]. Interestingly, a study on variants of concern analyzed host miRNA interactions with genomes of Wuhan-Hu-1, Beta, Delta, and Omicron variants. The study showed that Omicron exhibited a marked reduction in human miRNA binding sites compared with earlier variants, suggesting an increased ability to evade host miRNA-mediated antiviral regulation [97]. A clinical study revealed that SARS-CoV-2 infection induced miR-155 in the PBMCs of COVID-19 patients, which negatively correlated with *SOCS1*, and so in this case, immune and inflammatory responses increased [81]. Likewise, RSV, the most common viral cause of severe lower respiratory tract infections in infants, has been found to induce changes in host miRNAs involved in IFN signaling [98,99]. In particular, virus-induced miRNA remodeling contributed to viral replication through the inhibition of TLR4 signaling [80,100] and suppression of IFN receptor expression [78]. These observations indicate that IFN signaling constitutes a central regulatory hub repeatedly targeted by miRNA networks across respiratory RNA viruses. Despite considerable differences in viral biology, many pathogens exploit common miRNA-regulated mechanisms to modulate antiviral responses and optimize replication.

### 3.2. NF-κB Pathway, Apoptosis, and Autophagy

NF-κB signaling represents another major pathway frequently targeted during respiratory RNA virus infections. As a master regulator of inflammatory responses, NF-κB controls the expression of cytokines, chemokines, adhesion molecules, and numerous immune-related genes. Appropriate activation of this pathway is essential for pathogen clearance; however, excessive or prolonged NF-κB signaling may contribute to immunopathology and tissue damage [101]. Host miRNAs play an important role in regulating NF-κB activity through targeting key signaling intermediates, including *IRAK1*, *TRAF6*, and other components of innate immune pathways. Among the most extensively studied examples are miR-155 and miR-146a, which participate in the feedback regulation of inflammatory responses. While miR-155 induction is often associated with the amplification of inflammatory and antiviral responses, miR-146a generally acts as a negative regulator of NF-κB signaling [102,103]. Under physiological conditions, these feedback loops contribute to the resolution of inflammation and maintenance of immune homeostasis. However, across different respiratory RNA virus infections, these miRNA-regulated inflammatory circuits have been described to be modulated or subverted by viruses to dampen antiviral signaling and establish a more permissive intracellular environment. Consequently, the miRNA-mediated regulation of NF-κB signaling contributes not only to antiviral defense but also to immune evasion and infection-associated immunopathology [104]. Regarding IAV infection, an increased expression of miR-155 has been shown to contribute to the development of lethal ARDS in a murine model of infection [87]. Similarly, in SARS-CoV-2 infections, the altered expression of some miRNAs has been associated with severe inflammatory manifestations [105]; a high level of miR-155 has been measured in plasma from COVID-19 patients [81,105], as well as miR-200, leading to their proposal as potential biomarkers [106]. On the other hand, some of these miRNAs, in particular miR-155, have been explored as potential therapeutic targets, such as the anti-miR-155-attenuated lung cytokine storm induced by SARS-CoV-2 infection [88]. The NF-κB pathway is also closely linked to apoptosis. As a host defense mechanism, many viruses benefit from delaying or suppressing programmed cell death during the early stages of infection. On the other hand, the induction of apoptosis in the late stages of infection can promote cellular fragmentation and the release and spread of newly formed viral particles. Host miRNAs contribute significantly to this process by regulating both pro-apoptotic and anti-apoptotic factors, thereby influencing cell fate decisions during infection [69]. It has been shown that influenza virus-induced miR-29c mediated antiapoptotic-factor *BCL2L2* suppression, thus contributing to viral-induced apoptosis in epithelial cells [82]. Another study demonstrated that IAV increased, through miR-34a downregulation, pro-apoptotic *BAX* expression, suggesting another mechanism of influenza virus-induced cell death [83]. Increasing evidence indicates that host miRNAs play important roles in regulating another process for a long time considered a form of cell death, that is autophagy [107]. Autophagy is an evolutionarily conserved pathway involved in the degradation and recycling of intracellular components. During viral infection, autophagy can contribute to antiviral defense by promoting the degradation of viral components and facilitating immune signaling. However, numerous viruses have evolved strategies to manipulate autophagic pathways for their own benefit [108]. MiRNAs regulate multiple components of autophagy machinery and can therefore influence the balance between antiviral activity and viral exploitation [107]. Recently, it has been shown that RSV-induced exosome miRNA let-7i-5p acted as an autophagy inhibitor and, as a consequence, airway inflammation and asthmatic reaction were alleviated [109]. In parallel, viral replication imposes substantial metabolic demands on infected cells. To sustain productive infection, viruses frequently induce metabolic reprogramming involving glucose utilization, mitochondrial function, lipid synthesis, and cholesterol metabolism. Host miRNAs have emerged as key regulators of these pathways, linking metabolic homeostasis to antiviral defense. For example, miR-342-5p is an interferon-regulated miRNA that exerts broad-spectrum antiviral activity through the coordinated modulation of sterol biosynthesis. miR-342-5p acts at multiple levels in the sterol pathway, including the regulation of SREBP2-dependent mechanisms and direct targeting of some sterol-related genes, restricting the replication of unrelated viruses, including IAV [84]. A conceptually related mechanism has recently been described for miR-185 during coronavirus infection. Experimental overexpression of miR-185 restricted the entry and propagation of SARS-CoV-2, including several Spike variants, and reduced HCoV-229E replication. These effects were associated with a decreased expression of genes involved in lipid uptake and biosynthesis, together with altered *ACE2* expression. Thus, miR-185 appears to establish a less permissive lipid microenvironment by simultaneously affecting viral entry and host metabolic pathways required for coronavirus propagation [85]. Therefore, metabolic regulation represents an additional layer of miRNA-mediated antiviral defense. Despite employing distinct molecular strategies, respiratory RNA viruses frequently converge on shared miRNA-regulated host pathways, including IFN signaling, NF-κB-mediated inflammation, apoptosis, autophagy, and metabolic homeostasis. This functional convergence highlights the role of host miRNAs as context-dependent integrators of antiviral defense and viral adaptation [39,70].

### 3.3. Redox Homeostasis

Redox signaling represents additional regulatory interfaces in host–virus interactions. Although still limited, a growing body of evidence indicates the role of host miRNAs in the modulation of this signaling pathway also [110]. Respiratory RNA viruses remodel the intracellular redox environment and IAV provides a well-characterized example of such redox remodeling, by altering cellular antioxidant systems and promoting reactive oxygen species (ROS) accumulation [111]. For instance, the IAV-mediated downregulation of glucose-6-phosphate dehydrogenase (G6PD) impairs NADPH and glutathione homeostasis, generating intracellular pro-oxidant conditions that favor viral replication [112]. Moreover, while physiological levels of ROS function as signaling molecules involved in antiviral immunity and inflammatory responses, their excessive production can disrupt redox homeostasis, promoting cellular damage and contributing to viral pathogenesis, as demonstrated by the involvement of NADPH Oxidase 2 (Nox2)-derived ROS in IAV-induced pulmonary inflammation and injury [113] or in SARS-CoV-2 complications [114]. Although still few, some studies have established a link between miRNA and respiratory virus-induced redox remodeling: the miR-34c-5p/TLR5 axis regulated IAV-induced oxidative stress and inflammatory response in vitro [115]. Increased levels of miR-21/let-7b were measured in platelet-derived extracellular vesicles (pEVs) from COVID-19 patients and were associated with disease severity; in the same study, the authors showed that miR-21 carried by pEVs interacted with TLR7/8 in neutrophils, resulting in Nox activation to promote ROS production and neutrophil extracellular trap (NET) enhancement [116]. In another study, miR-144 was significantly increased in plasma from severe COVID-19 patients and negatively correlated with nuclear factor erythroid 2-related factor 2 (Nrf2) protein concentration, suggesting an interference of miR-144 with the Nrf2 antioxidant signaling pathway in these patients [117].

So, within this redox-altered environment, miRNAs may function both as sensors and regulators of oxidative stress. Indeed, ROS-dependent signaling can modify miRNA expression profiles, whereas miRNAs can in turn regulate ROS-generating systems such as Nox, as well as antioxidant responses, including the Nrf2 pathway, establishing bidirectional regulatory circuits between miRNA networks and cellular redox homeostasis [110,118,119]. The biological consequences of this crosstalk can extend beyond the control of oxidative damage. Redox imbalance can influence antiviral and inflammatory signaling pathways, including NF-κB activity and inflammasome activation. In particular, excessive ROS accumulation can promote NLRP3 inflammasome activation, while persistent reciprocal activation of oxidative stress and inflammasome-driven inflammation may contribute to an OxInflammatory state associated with tissue damage and disease progression [120]. Taken together, these observations identify redox homeostasis as another point of convergence between respiratory RNA viruses and host miRNA networks. By connecting ROS production, antioxidant defenses, innate immune signaling, and inflammatory responses, miRNAs may contribute to determining whether redox remodeling supports effective antiviral defense or instead favors viral replication and infection-associated immunopathology, even if data are still limited and specific targets and mechanisms need to be identified and characterized.

### 3.4. miRNAs and Immunopathology

Host miRNAs are increasingly recognized as important determinants of infection-associated immunopathology. Because individual miRNAs can simultaneously regulate multiple components of innate and adaptive immunity, they occupy a critical position at the interface between protective antiviral responses and pathological inflammation [104]. Rather than functioning as simple on–off switches, miRNAs frequently act as molecular rheostats that shape both the amplitude and kinetics of immune activation. By regulating PRR pathways, IFN responses, JAK/STAT signaling, NF-κB activation, and cytokine production, miRNAs participate in feedback and feed-forward circuits that coordinate antiviral defense with the resolution of inflammation. Disruption of these circuits may therefore produce two opposite but equally detrimental outcomes: insufficient antiviral immunity or uncontrolled immune activation, leading to tissue injury [104]. miR-146a and miR-155 illustrate this context-dependent functional duality. miR-146a is induced downstream of TLRs and cytokine signaling and functions as a negative-feedback regulator by targeting the signaling intermediates *IRAK1* and *TRAF6*, thereby restraining NF-κB-dependent inflammatory responses [121]. During viral infection, however, this regulatory activity may have different consequences depending on the cellular and infectious context. Although miR-146a can protect tissues by limiting excessive inflammation, its sustained induction may attenuate antiviral signaling and create conditions favorable to viral persistence or replication [122]. Conversely, miR-155 is an important regulator of immune-cell activation and inflammatory cytokine production. Its induction can support antimicrobial and antiviral responses, whereas excessive or persistent miR-155 activity has been associated with sustained inflammation and immune-mediated tissue damage [123], as in ARDS induced by IAV [87], or during infant RSV infection [124]. Thus, the biological effect of a miRNA cannot be inferred solely from its classification as pro- or anti-inflammatory but depends on its cell source, target availability, abundance, and timing during infection. This regulatory imbalance is particularly relevant in severe respiratory viral disease. Aberrant miRNA expression has been associated with hyperinflammation, epithelial and endothelial dysfunction, immune-cell dysregulation, acute lung injury, and acute respiratory distress syndrome [125]. In this setting, miRNAs may act locally, influencing cytokine release, leukocyte recruitment, cell death, vascular permeability, and tissue repair. The resulting immunopathology is therefore not simply a consequence of increased cytokine concentrations but rather reflects the failure of coordinated regulatory networks to terminate inflammation and restore pulmonary homeostasis [126]. Recent clinical evidence has identified miR-9 as a potential component of this dysregulated inflammatory network in COVID-19 patients. Circulating miR-9 levels were significantly elevated in patients with COVID-19 compared with healthy controls, with particularly high levels observed in non-survivors. miR-9 levels showed a strong positive correlation with NF-κB, which, in turn, correlated with the pro-inflammatory cytokines IL-6, IL-1β, and TNF-α. These findings associate miR-9 dysregulation with enhanced NF-κB-mediated inflammation and adverse clinical outcomes [127]. MiR-26a-5p, miR-29b-3p, and miR-34a-5p were identified as regulators of mRNA targets involved in endothelial dysfunction and their deregulation was associated with the occurrence of severe lung injury and immunothrombosis in COVID-19 patients [128]. McDonald et al. (2021) [86] identified miR-2392 as a host miRNA induced during SARS-CoV-2 infection that suppresses mitochondrial gene expression while promoting glycolysis, hypoxia-associated responses, and inflammatory signaling. Inhibition of miR-2392 reduced viral burden and reversed several infection-associated transcriptional alterations. These observations suggest that selected miRNAs may simultaneously support viral replication and contribute to the inflammatory and metabolic phenotype associated with severe disease. Clinical observations further support the relevance of miRNA-mediated feedback regulation to disease outcome and treatment response. Reduced circulating levels of miR-146a-5p were associated with a clinical non-response to the IL-6 receptor inhibitor tocilizumab in patients with COVID-19. This observation is consistent with an impaired negative-feedback mechanism controlling inflammatory signaling. Nevertheless, the association does not establish miR-146a-5p as a direct determinant of treatment response and requires validation in larger and independent patient cohorts [129]. In addition to acting intracellularly, miRNAs can be released into the extracellular environment either bound to proteins or enclosed within extracellular vesicles. Vesicle-associated miRNAs can potentially be transferred among epithelial and endothelial cells, macrophages, and other immune populations, thereby extending regulatory signals beyond the initially infected cell. Distinct extracellular-vesicle miRNA profiles have been detected in patients with COVID-19 pneumonia and ARDS, with predicted targets enriched in immune, inflammatory, and coagulation-related pathways. However, whether extracellular-vesicle-associated miRNAs actively propagate systemic immunopathology or primarily represent biomarkers of cellular activation and tissue injury remains incompletely resolved [130]. Increasing evidence also indicates that disease severity is accompanied by distinct circulating miRNA signatures. Garcia-Giralt et al. (2022) [131] identified extensive differences in circulating miRNA profiles between patients with COVID-19-associated ARDS requiring mechanical ventilation and patients with less severe disease. Among the validated candidates, miR-369-3p was significantly reduced in patients requiring mechanical ventilation, suggesting a potential association with severe inflammatory lung injury. More recently, profiling of plasma-derived extracellular vesicles revealed extensive miRNA dysregulation in patients with severe COVID-19. Among the differentially expressed candidates, miR-1469 and miR-6124 emerged as potential predictors of mortality. Although these findings support the prognostic potential of extracellular-vesicle-associated miRNAs, their clinical utility requires prospective validation in larger and more heterogeneous populations [132]. The interpretation of circulating miRNA signatures nevertheless presents several challenges. Differences in biological specimen, extracellular-vesicle isolation, sampling time, disease stage, treatment, age, and comorbidities can substantially influence the detected miRNA profiles. Moreover, changes in circulating miRNA abundance may reflect active secretion, immune-cell activation, altered tissue composition, or passive release from injured and dying cells. Consequently, association with disease severity does not necessarily establish a functional contribution to immunopathology [130,131,132]. Importantly, the involvement of miRNAs in immunopathology highlights their context-dependent duality during infection. Regulatory mechanisms that protect pulmonary tissues from excessive inflammation may suppress antiviral immunity if activated too early or too strongly. Conversely, miRNAs that enhance pathogen recognition and cytokine production may facilitate viral clearance but increase collateral tissue injury when their expression persists. Cell-type-specific and longitudinal analyses are therefore essential to distinguish protective miRNA responses from those that promote disease [104,122,123]. Taken together, host miRNAs influence not only viral replication but also the transition from protective immunity to pathological inflammation. By coordinating cytokine production, immune-cell activation, cellular metabolism, intercellular communication, and tissue repair, they contribute to determining the clinical outcome of respiratory viral infections [19,104]. Their stability in circulation and association with disease severity make them attractive diagnostic and prognostic candidates, although their broad, dynamic, and context-dependent target repertoires represent both an opportunity and a challenge for therapeutic intervention [131,132].

## 4. Do RNA Viruses Encode Their Own miRNAs?

The possibility that RNA viruses encode functional miRNAs remains one of the most debated issues in the field of virus–miRNA interactions. While virus-encoded miRNAs are well established for several DNA viruses, particularly herpesviruses, their existence and biological relevance in RNA viruses are far less clear [133]. This controversy arises from both biological constraints and technical limitations. Unlike DNA viruses, many RNA viruses use their genomic RNA directly as a replication template or messenger RNA; therefore, the processing of viral hairpin structures by Drosha or Dicer could potentially damage the viral genome or generate double-stranded RNA intermediates capable of activating antiviral defenses [9,10]. For this reason, RNA viruses are generally thought to rely predominantly on the modulation of host miRNAs rather than on the production of canonical viral miRNAs or on the production of non-canonical miRNAs [134].

### 4.1. Established Evidence from DNA Viruses

DNA viruses provide the clearest and most extensively validated examples of virus-encoded miRNAs. Herpesviruses, including HSV-1 and -2, HCMV, Epstein–Barr virus (EBV), and Kaposi’s sarcoma-associated herpesvirus (KSHV), encode multiple viral miRNAs that regulate both viral and host gene expression [135]. These miRNAs contribute to viral persistence, latency, immune evasion, apoptosis control, and, in some cases, tumorigenesis. Because DNA viruses replicate in cellular compartments where access to canonical miRNA biogenesis machinery is compatible with their life cycle, the production of viral miRNAs represents an efficient strategy to fine-tune gene expression without generating highly immunogenic proteins. This situation contrasts with that of RNA viruses, for which the production of canonical miRNAs is biologically more problematic. The generation of a mature miRNA typically requires the processing of structured RNA precursors. In the context of an RNA virus, such processing could interfere with genome integrity, replication, translation, or packaging. These constraints explain why authentic viral miRNAs are much less common, and much more controversial, among RNA viruses [134].

### 4.2. RNA Viruses and Controversial Evidence

Several studies have reported the presence of small RNA species originating from RNA virus genomes [133,136,137]. However, whether these molecules should be considered authentic viral miRNAs remains uncertain. A key distinction must be made between canonical viral miRNAs, viral miRNA-like small RNAs, and virus-derived siRNAs (Table 2). Canonical viral miRNAs are expected to arise from defined hairpin precursors and to function through RISC-dependent target repression. By contrast, viral miRNA-like RNAs may resemble miRNAs in size or function but follow non-canonical biogenesis routes. Virus-derived siRNAs, instead, are typically generated from double-stranded viral RNA intermediates and belong to antiviral RNA interference pathways rather than to classical miRNA biology. One of the most discussed examples is the H5N1 influenza virus-derived miRNA-like small RNA miR-HA-3p [138]. This small RNA was reported to originate from a stem–loop-containing region of the viral HA segment and to enhance cytokine production during H5N1 infection by targeting the poly(rC)-binding protein 2 (PCBP2). Functionally, miR-HA-3p was linked to increased inflammatory responses and mortality in experimental models, suggesting that an RNA virus-derived miRNA-like molecule may contribute to virulence and immunopathology. Nevertheless, this molecule is generally described as “miRNA-like” rather than as a fully canonical viral miRNA, reflecting the continuing uncertainty regarding its biogenesis and generalizability. Pawlica et al. (2021) [139] discovered a miRNA-like small RNA expressed by SARS-CoV-2, named CoV2-miR-O7a, for its origin from the viral Open Reading Frame (ORF)7a, which was associated with AGO proteins and components of the RNA interference pathway. So, CoV2-miR-O7a production relied on cellular machinery, but was independent of the Drosha protein. The authors also identified its putative target in interferon signaling factors. Studies on RSV and another closely related member of the *Pneumoviridae* family, human metapneumovirus, have identified small RNA derived from the latter, in the cytoplasm, suggesting that, also in this case, they are generated through a non-canonical pathway [140]. Dengue virus provides another frequently cited example. A Dengue virus 2-derived small RNA, DENV-vsRNA-5, has been reported to display miRNA-like properties and to autoregulate viral replication by targeting viral RNA [141]. This finding supports the possibility that some RNA viruses can generate functional small RNAs with regulatory activity. However, this evidence also highlights the difficulty of defining these molecules as canonical miRNAs, particularly because their production may depend on cell type, host species, viral replication stage, and the experimental system used. Moreover, many RNA viruses appear to have evolved strategies to avoid extensive engagement with the host miRNA machinery, possibly to prevent restriction by endogenous miRNAs or deleterious cleavage of viral RNA [142]. These observations support the view that, although RNA virus-derived miRNA-like small RNAs may exist in specific contexts, canonical viral miRNA production is unlikely to represent a broadly conserved strategy among RNA viruses.

### 4.3. Technical Limitations and Current State of the Debate

Several methodological challenges contribute to the ongoing debate [143]. First, small RNA sequencing can detect short viral RNA fragments, but the mere presence of a small RNA species is not sufficient to define it as a functional miRNA. Authentic miRNA assignment requires evidence of precise processing, defined precursor structure, association with Argonaute proteins, reproducible expression, and sequence-specific repression of biologically relevant targets. Second, distinguishing viral miRNAs from degradation products remains difficult. During active viral replication, large amounts of viral RNA are produced and degraded, generating small fragments that may resemble miRNAs in size but lack regulatory function. Without rigorous validation, such fragments may be misinterpreted as miRNA-like molecules. Third, the biogenesis of RNA virus-derived small RNAs may be non-canonical. Some reported viral miRNA-like RNAs do not appear to follow the classical Drosha–Dicer pathway and may instead arise through alternative processing mechanisms. While this does not exclude biological activity, it complicates their classification as true viral miRNAs. Finally, the biological relevance of RNA virus-derived small RNAs remains uncertain. Even when such molecules are detected and shown to have regulatory potential, it is often unclear whether they are produced at sufficient levels during natural infection to influence viral replication, immune responses, or disease outcome [143]. Overall, the current evidence supports a cautious interpretation. RNA viruses may, under specific conditions, generate small regulatory RNAs with miRNA-like properties. However, the major biological relevance of miRNA pathways in RNA virus infections appears to lie in the manipulation of host miRNAs and host miRNA machinery rather than in the widespread production of authentic viral miRNAs. This distinction is particularly important for respiratory RNA viruses, where host miRNA remodeling provides a robust and recurrent mechanism for regulating antiviral immunity, inflammation, and viral fitness.

## 5. Diagnostic and Therapeutic Potential of miRNAs

The central role of miRNAs in regulating antiviral immunity, inflammation, and host–virus interactions has generated considerable interest in their clinical application. Because alterations in miRNA expression occur early during infection and reflect both viral activity and host responses, miRNAs have emerged as promising candidates for diagnostic biomarkers and therapeutic targets. Unlike conventional protein biomarkers, miRNAs are remarkably stable in biological fluids, can be detected using minimally invasive procedures, and often reflect specific pathological processes [144,145]. Consequently, miRNA-based approaches are increasingly being explored for disease diagnosis, prognostic stratification, and therapeutic intervention in respiratory viral infections [146].

### 5.1. miRNAs as Biomarkers

The identification of reliable biomarkers capable of predicting disease severity, monitoring disease progression, and guiding therapeutic decisions remains a major challenge in respiratory viral infections. In recent years, circulating miRNAs have attracted considerable attention as potential biomarkers because of their stability in serum, plasma, saliva, and other biological fluids [147,148]. Encapsulation within extracellular vesicles (EVs), association with RNA-binding proteins, and incorporation into lipoprotein complexes protect miRNAs from degradation and facilitate their detection in clinical samples [149]. Numerous studies have demonstrated that viral infections induce characteristic changes in host miRNA expression profiles and particular interest has emerged in the context of severe respiratory infections, where dysregulated miRNA expression has been associated with hyperinflammation, acute lung injury, and ARDS. In COVID-19, several circulating and EV-associated miRNAs have been linked to disease severity, clinical outcome, and recently, to long-COVID-19 sequelae [19,132,150,151]. Similarly, altered miRNA profiles have been reported in severe IAV and RSV infections, where specific miRNAs correlate with cytokine production and markers of systemic inflammation, suggesting potential utility for risk stratification and prognostic assessment [152]. Beyond disease severity, miRNA signatures may also provide information regarding treatment response and disease progression. The integration of miRNA profiling with conventional clinical and laboratory parameters could therefore improve patient stratification and facilitate the development of personalized therapeutic approaches. Despite their promise, the translation of miRNA biomarkers into routine clinical practice remains challenging. Variability in sample collection, detection methods, normalization strategies, and patient populations have limited reproducibility across studies. Nevertheless, advances in high-throughput sequencing technologies and standardized analytical pipelines are expected to improve the reliability and clinical utility of miRNA-based diagnostics [153].

### 5.2. Therapeutic Applications: miRNA Mimics and Inhibitors (AntagomiRs)

The ability of miRNAs to simultaneously regulate multiple genes within interconnected biological pathways has stimulated considerable interest in their therapeutic exploitation. Unlike conventional, direct antivirals that target specific viral proteins, miRNA-based therapies have the potential to modulate host pathways involved in viral replication, immune responses, and inflammation. This host-directed approach may provide broader antiviral activity and reduce the likelihood of resistance associated with viral mutation [154]. Two principal therapeutic strategies have been explored: the restoration of beneficial antiviral miRNAs through miRNA mimics and the inhibition of pathogenic or pro-viral miRNAs through antisense oligonucleotides, commonly referred to as antagomiRs. MiRNA mimics are synthetic molecules designed to restore or enhance the activity of endogenous miRNAs with antiviral or immunoregulatory functions. This approach is particularly attractive when protective miRNAs are downregulated during infection. By re-establishing physiological regulatory networks, miRNA mimics may strengthen antiviral defenses and limit viral replication [144]. Several experimental studies have demonstrated the therapeutic potential of antiviral miRNAs. Administration of agomir-delivered antiviral miRNAs has been shown to suppress IAV replication and improve survival in animal models [16]. Among candidate antiviral miRNAs, miR-26a has attracted particular attention because of its ability to enhance RIG-I-mediated type I interferon responses through targeting *USP15* [47]. Importantly, miR-26a exhibits broad-spectrum antiviral activity against multiple RNA viruses, suggesting that the modulation of host antiviral pathways may provide protection that extends beyond a single viral species [47,48]. These findings support the concept that the restoration of endogenous antiviral miRNAs may represent a promising host-directed therapeutic strategy capable of complementing conventional antiviral treatments. An alternative therapeutic strategy involves the inhibition of miRNAs that promote viral replication or contribute to excessive inflammation. AntagomiRs are chemically modified antisense oligonucleotides that bind target miRNAs and prevent their interaction with endogenous mRNA targets [155]. This approach may be particularly useful when viral infection induces host miRNAs that suppress IFN signaling or facilitate immune evasion. Likewise, the inhibition of miRNAs involved in hyperinflammatory responses may help reduce tissue damage and improve clinical outcomes during severe respiratory infections [11]. Because many pathogenic processes are mediated by complex regulatory networks rather than single genes, targeting upstream miRNA regulators offers an attractive strategy for simultaneously modulating multiple disease-associated pathways. This system-level approach may prove especially valuable for respiratory RNA viruses, where disease severity often results from dysregulated host responses rather than direct viral cytopathic effects alone. However, despite encouraging preclinical findings, miRNA-based therapeutics for respiratory RNA virus infections have not yet received a clinical evaluation. To our knowledge, no interventional clinical trials have evaluated miRNA mimics or inhibitors specifically against IAV, SARS-CoV-2, or RSV. Clinical proof of concept for host miRNA targeting in viral infections has been provided for chronic hepatitis C, where miravirsen, an antisense inhibitor of host miR-122, produced a dose-dependent reduction in HCV RNA [156]. Experience with miRNA therapeutics for other diseases, such as cancer, including the termination of the MRX34 miR-34a mimic trial following severe immune-mediated adverse events, underscores the challenges associated with pleiotropic target regulation, immunogenicity, and off-target toxicity [157]. Thus, the therapeutic application of miRNA modulation in respiratory viral infections should currently be considered preclinical.

#### Delivery Strategies

Efficient and safe delivery remains one of the principal obstacles to the clinical implementation of miRNA therapeutics. Naked RNA molecules are rapidly degraded in biological fluids and exhibit limited cellular uptake, necessitating the development of specialized delivery platforms. Several approaches have been investigated, including chemically modified oligonucleotides, viral vectors, lipid nanoparticles (LNPs), and extracellular vesicles [148]. Among these, LNP-based systems have gained particular attention following their successful application in nucleic acid therapeutics and mRNA vaccines. Extracellular vesicles represent an additional promising platform because of their natural biocompatibility, low immunogenicity, and intrinsic capacity for intercellular communication. Their ability to transport RNA cargoes across biological barriers has generated considerable interest in the EV-mediated delivery of therapeutic miRNAs [158]. For respiratory viral infections, local administration through intranasal delivery offers a particularly attractive strategy. By targeting the primary site of infection, intranasal delivery may increase therapeutic concentrations in respiratory tissues while minimizing systemic exposure and off-target effects. Experimental studies using intranasal administration of miRNA-based therapeutics have already demonstrated encouraging antiviral effects in preclinical models [16].

### 5.3. Current Limitations and Challenges

Despite encouraging experimental results, several challenges continue to hinder the clinical translation of miRNA-based diagnostics and therapeutics. A major concern is the pleiotropic nature of miRNA regulation. Because a single miRNA may influence hundreds of target transcripts, therapeutic modulation carries an inherent risk of biological off-target responses [155]. The effects of miRNA modulation may also be difficult to predict across different biological contexts. The activity of an individual miRNA depends on target abundance, cell type, infection stage, and the surrounding regulatory network [153,154,155]. Moreover, inter-individual differences in baseline miRNA expression, age, immune status, comorbidities, and disease severity may influence both therapeutic efficacy and safety [146,153,154]. These factors of variability limit the extrapolation of findings from cell culture and animal models to humans and complicate the selection of therapeutic candidates, dosing regimens, and treatment windows [149,154,155,158].

Clinical experience in other disease settings further illustrates both the potential and the limitations of miRNA-based interventions. As mentioned before, in patients with chronic HCV infection, the inhibition of miR-122 by miravirsen produced dose-dependent and prolonged reductions in viral RNA, providing clinical proof of concept for host miRNA targeting. However, undetectable HCV RNA was experienced in only a subset of patients, and virological rebound occurred in several participants after treatment discontinuation [156]. Regarding the liposomal miR-34a mimic, MRX34, the phase I trial demonstrated target engagement and antitumor activity but was terminated early following serious immune-mediated adverse events associated with four patient deaths [157]. Although these studies did not involve respiratory viral infections, they show that molecular target engagement does not necessarily translate into sustained efficacy or acceptable safety.

Delivery remains another significant obstacle. Achieving efficient, tissue-specific, and sustained delivery while avoiding toxicity and immunogenicity is essential for successful therapeutic development [148]. The dynamic and context-dependent nature of miRNA expression also complicates both biomarker discovery and therapeutic targeting. Future progress will likely depend on the integration of miRNA profiling with other molecular, immunological, and clinical datasets. Advances in RNA delivery technologies, artificial intelligence-assisted biomarker discovery, and systems biology approaches may facilitate the development of more precise and personalized miRNA-based interventions [159]. Although significant challenges remain, the expanding understanding of miRNA biology suggests that these molecules may ultimately become valuable tools for both diagnosis and treatment of respiratory viral diseases.

## 6. Conclusions and Future Perspectives

Host miRNAs have emerged as central regulators of respiratory RNA virus infections, functioning at the intersection of antiviral immunity, inflammatory responses, and viral adaptation. MiRNAs actively shape the outcome of infection by controlling gene networks involved in viral sensing, IFN signaling, apoptosis, autophagy, and cell homeostasis. The ability of viruses to manipulate host miRNA expression and, in some cases, components of the miRNA biogenesis machinery, highlights the importance of these regulatory pathways in virus–host interactions. A recurring theme throughout this review is the remarkable convergence of diverse respiratory RNA viruses on a limited number of miRNA-regulated host pathways. Despite substantial differences in genome organization, replication strategies, and pathogenic potential, viruses such as IAV, SARS-CoV-2, and RSV repeatedly target common regulatory hubs, including IFN signaling, NF-κB-mediated inflammatory responses, apoptosis, and cellular metabolic pathways. This convergence underscores the evolutionary advantage of exploiting highly conserved host networks and reinforces the concept that miRNAs serve as critical integrators of antiviral defense and, on the other hand, of viral replication. At the same time, important questions remain unresolved. Among the most debated is whether RNA viruses broadly encode authentic viral miRNAs or instead generate non-canonical miRNA-like small RNAs under specific biological conditions. Although several intriguing examples have been reported, current evidence suggests that the modulation of host miRNA networks represents a far more prevalent and biologically significant strategy than the production of canonical viral miRNAs. Continued refinement of sequencing technologies, bioinformatic pipelines, and functional validation approaches will be essential for resolving this longstanding controversy. The growing recognition of miRNAs as regulators of both antiviral immunity and immunopathology also has important translational implications. MiRNA expression profiles have shown considerable promise as biomarkers of disease severity, prognosis, and treatment response, while therapeutic approaches based on miRNA mimics, antagomiRs, and RNA delivery platforms continue to advance. Nevertheless, challenges related to specificity, delivery, safety, and reproducibility must be addressed before miRNA-based strategies can be broadly implemented in clinical practice. Looking forward, future research should focus on integrating miRNA biology with system-level analyses of host–pathogen interactions. Combining transcriptomic, epigenetic, immunological, and clinical datasets will provide a more comprehensive understanding of how miRNA networks coordinate antiviral responses and disease progression. Such approaches may ultimately facilitate the development of novel host-directed therapies capable of simultaneously limiting viral replication and mitigating excessive inflammatory responses. In conclusion, miRNAs represent a critical layer of regulation in respiratory RNA virus infections and provide a unifying framework through which many aspects of viral pathogenesis can be interpreted. Further investigation of miRNA-mediated mechanisms will not only improve our understanding of virus–host interactions but may also contribute to the development of innovative diagnostic and therapeutic strategies for current and emerging respiratory viral diseases.

## Figures and Tables

**Table 1:** Host miRNA-mediated regulation of cellular pathways during respiratory RNA virus infection.

Respiratory Virus	Host miRNA	Reported Target(s)	Main Host Pathway	Functional Role	Reported Biological Effect	Ref.
IAV	miR-9-1	*TNKS1*	Type I IFN signaling	Antiviral	*TNKS1* repression enhanced type I IFN production and STAT1 signaling, reduced viral replication, and increased resistance to IAV infection.	[72]
IAV	miR-206	*TNKS2*	Type I IFN signaling	Antiviral	*TNKS2* repression activated JNK/c-Jun and IFN-I signaling and inhibited IAV replication.	[73]
IAV	miR-302 cluster	IRF family members, mainly *IRF1* and *IRF2*; *IRF1AS* as an indirect downstream effector	IRF/IFN signaling and enhancer-associated transcription	Antiviral; species-dependent	Regulation of the *IRF–* *IRF1AS* circuit promoted antiviral hub genes and lncRNAs, most of which inhibited IAV replication.	[74]
IAV	miR-146a	*TRAF6*	Type I IFN signaling	Pro-viral	Attenuation of the antiviral response and enhancement of IAV replication.	[75]
IAV	miR-200c	*CNTN1*; MAVS as an indirect downstream mediator	RIG-I/MAVS signaling	Pro-viral	*CNTN1* repression accelerated MAVS degradation, weakened antiviral signaling, and facilitated IAV replication.	[76]
IAV	miR-221, downregulated during infection	*SOCS1*	Type I IFN signaling	Antiviral miRNA suppressed by IAV	miR-221 downregulation was associated with increased *SOCS1* expression and suppression of IFN-I response.	[77]
RSV	miR-29a, induced by viral NS1	*IFNAR1*	Type I IFN signaling	Pro-viral	*IFNAR1* repression impaired cellular responsiveness to IFN-I and promoted RSV replication.	[78]
IAV, H9N2 and H1N1	gga-miR-92	*TNFRSF1B*; TRAF3 as an indirect downstream mediator	Type I IFN signaling	Antiviral; virus- and host-cell-dependent	Direct repression of *TNFRSF1B* limited autophagolysosomal degradation of TRAF3, enhanced IFN-I signaling, and inhibited H9N2 and H1N1 replication in avian DF-1 cells. In human A549 cells, miR-92 inhibited H1N1 but not H9N2 replication.	[79]
RSV	miR-26b, upregulated during infection	*TLR4*	TLR4/NF-κB signaling	Pro-viral and immunomodulatory	*TLR4* repression impaired innate immune signaling and favored RSV infection.	[80]
SARS-CoV-2	miR-155, upregulated during infection	*SOCS1*, inversely associated; direct targeting not demonstrated in the clinical study	IFN and inflammatory signaling	Inflammation-associated; context-dependent	Increased miR-155 expression in PBMCs correlated inversely with *SOCS1* and was associated with enhanced immune and inflammatory responses.	[81]
IAV	miR-29c, induced during infection	*BCL2L2*	Apoptosis	Pro-apoptotic	*BCL2L2* repression promoted IAV-induced apoptosis in A549 epithelial cells.	[82]
IAV	miR-34a, downregulated during infection	*BAX*	Apoptosis	Anti-apoptotic under basal conditions	miR-34a downregulation increased *BAX* expression and contributed to IAV-induced apoptosis.	[83]
IAV, H1N1	miR-342-5p	*SREBF2* and multiple sterol-pathway genes	Sterol biosynthesis and metabolic homeostasis	Antiviral	Multihit inhibition of the sterol biosynthetic pathway restricted IAV replication.	[84]
SARS-CoV-2, HCoV-229E	miR-185	*SREBP2/SREBF, SCARB1* and *AGPAT3*; *SQLE* and *ACE2* indirectly reduced	Lipid metabolism and viral entry	Antiviral	Remodeling of the cellular lipid environment reduced SARS-CoV-2 entry and propagation and inhibited HCoV-229E replication and infectivity.	[85]
SARS-CoV-2	miR-2392, induced during infection	Mitochondrial gene network, including *OXPHOS*- and metabolism-related transcripts	Mitochondrial metabolism, glycolysis, hypoxia, and inflammation	Pro-viral and immunopathogenic	miR-2392 suppressed mitochondrial functions and promoted glycolytic, hypoxic, and inflammatory responses; its inhibition reduced viral burden in experimental models.	[86]
IAV, H1N1	miR-155-5p, upregulated in alveolar type II cells	—	NF-κB-mediated inflammation and lung injury	Immunopathogenic	Increased miR-155-5p expression contributed to severe pulmonary inflammation and lethal ARDS in infected mice.	[87]
SARS-CoV-2	miR-155	Multiple inflammatory regulators	Inflammatory cytokine signaling and lung injury	Immunopathogenic; potential therapeutic target	Anti-miR-155 treatment reduced pulmonary cytokine dysregulation and improved survival in SARS-CoV-2-infected mice.	[88]

The em dash (—) indicates that no specific molecular target was reported in the cited study.

**Table 2:** Main features distinguishing canonical viral miRNAs, viral miRNA-like small RNAs, and virus-derived siRNAs.

Features	Canonical Viral miRNAs	Viral miRNA-like Small RNAs	Virus-Derived siRNAs (vsiRNAs)
Origin	Defined viral hairpin precursor	Structured viral RNA region	Viral dsRNA intermediates
Biogenesis	Drosha–DGCR8- and Dicer-dependent; followed by AGO loading	Variable or incompletely defined; may involve AGO and bypass Drosha or Dicer	Dicer cleavage of viral dsRNA, followed by AGO loading; Drosha-independent
Molecular profile	~22 nt; precise 5′ end; defined mature/passenger-strand duplex	20–24 nt; precursor and processing signatures may be incomplete	21–24 nt; highly complementary duplexes mapping to viral RNA
Mode of action	Repression or destabilization of viral or host transcripts through RISC	Candidate-specific miRNA-like regulation of viral or host targets	Sequence-specific cleavage or silencing of viral RNA
Evidence required	Defined precursor, precise processing, AGO association, direct target validation, and functional evidence	Reproducible detection, exclusion of degradation products, processing, and target validation	Dicer dependence, AGO loading, siRNA-like duplexes, and antiviral activity
Examples/ current evidence	Common in DNA viruses; not conclusively demonstrated for IAV, SARS-CoV-2, or RSV	H5N1 miR-HA-3p; CoV2-miR-O7a; DENV-vsRNA-5	Well-established in plants and invertebrates; reported but debated in mammals
Main limitation	Size or predicted hairpin alone is insufficient for classification	Descriptive, mechanistically heterogeneous category	Viral degradation fragments may resemble authentic vsiRNAs

Note: Classification relies primarily on biogenesis and functional validation rather than viral origin or RNA length alone. AGO association is supportive but does not independently establish canonical miRNA identity. Abbreviations: AGO, Argonaute; DENV, Dengue virus; DGCR8, DiGeorge syndrome critical region 8; dsRNA, double-stranded RNA; IAV, influenza A virus; RISC, RNA-induced silencing complex; RSV, respiratory syncytial virus; vsiRNA, virus-derived small interfering RNA.

## Data Availability

No new data were created or analyzed in this study. Data sharing is not applicable to this article.

## Abbreviations

The following abbreviations are used in this manuscript:

ACE2	Angiotensin-converting enzyme 2
ARDS	Acute respiratory distress syndrome
EBV	Epstein–Barr virus
EV	Extracellular vesicle
G6PD	Glucose-6-phosphate dehydrogenase
HCMV	Human cytomegalovirus
HSV	Herpes simplex virus
IAV	Influenza A virus
IFN	Interferon
IFN-I	Type I interferon
IRF	Interferon regulatory factor
ISG	Interferon-stimulated gene
KSHV	Kaposi’s sarcoma-associated herpesvirus
LNP	Lipid nanoparticle
mRNA	Messenger RNA
miRNA	microRNA
NET	Neutrophil extracellular trap
Nox	NADPH oxidase
Nrf2	Nuclear factor erythroid 2-related factor 2
PAMP	Pathogen-associated molecular pattern
pre-miRNA	Precursor miRNA
pri-miRNA	Primary transcript miRNA
PRR	Pattern-recognition receptor
RIG-I	Retinoic acid-inducible gene-I
ROS	Reactive oxygen species
RSV	Respiratory syncytial virus
SARS-CoV-2	Severe acute respiratory syndrome coronavirus 2
TGF	Transforming growth factor
TLR	Toll-like receptors
TRBP	TAR RNA-binding protein
3′-UTR	3′-untranslated region
VSV	Vesicular stomatitis virus
